# Injury and immune response: applying the danger theory to mosquitoes

**DOI:** 10.3389/fpls.2014.00451

**Published:** 2014-09-09

**Authors:** Miguel Moreno-García, Benito Recio-Tótoro, Fabiola Claudio-Piedras, Humberto Lanz-Mendoza

**Affiliations:** ^1^Centro de Investigaciones Sobre Enfermedades Infecciosas, Instituto Nacional de Salud PúblicaCuernavaca, México; ^2^Instituto de Biotecnología, Posgrado en Ciencias Bioquímicas, Universidad Nacional Autónoma de MéxicoCuernavaca, México; ^3^Facultad de Medicina, Posgrado en Ciencias Biológicas, Universidad Nacional Autónoma de MéxicoMéxico City, México

**Keywords:** danger/damage, immune response, insects, mosquitoes, wound

## Abstract

The insect immune response can be activated by the recognition of both non-self and molecular by-products of tissue damage. Since pathogens and tissue damage usually arise at the same time during infection, the specific mechanisms of the immune response to microorganisms, and to tissue damage have not been unraveled. Consequently, some aspects of damage caused by microorganisms in vector-borne arthropods have been neglected. We herein reassess the *Anopheles–Plasmodium* interaction, incorporating Matzinger’s danger/damage hypothesis and George Salt’s injury assumptions. The invasive forms of the parasite cross the peritrophic matrix and midgut epithelia to reach the basal lamina and differentiate into an oocyst. The sporozoites produced in the oocyst are released into the hemolymph, and from there enter the salivary gland. During parasite development, wounds to midgut tissue and the basement membrane are produced. We describe the response of the different compartments where the parasite interacts with the mosquito. In the midgut, the response includes the expression of antimicrobial peptides, production of reactive oxygen species, and possible activation of midgut regenerative cells. In the basal membrane, wound repair mainly involves the production of molecules and the recruitment of hemocytes. We discuss the susceptibility to damage in tissues, and how the place and degree of damage may influence the differential response and the expression of damage associated molecular patterns (DAMPs). Knowledge about damage caused by parasites may lead to a deeper understanding of the relevance of tissue damage and the immune response it generates, as well as the origins and progression of infection in this insect–parasite interaction.

## INTRODUCTION

In the last 30 years research into insect immunity has blossomed, providing overwhelming evidence of the ecological costs of the immune response. It is now known that insect immunity is intimately involved in the evolution of diverse survival and reproductive strategies and correlated traits (immunity vs. developmental rates, egg production, coloring, etc.). There has also been rapid progress in unraveling the mechanisms of insect immune responses and their effects on pathogens. The distinction between self and non-self, between endogenous and exogenous, which is a fundamental aspect of vertebrate immunity, also seems to be pivotal for insect immunity (Klein, 1999). Insects, like vertebrates, are in contact with many microorganisms. Whereas some of these interactions do not have a detrimental effect on the host, others do. Hence, the concept of pathogen-associated molecular patterns (PAMPs), proposed by (Medzhitov and Janeway, 2002), has been extended to microorganism-associated molecular patterns (MAMPs) introduced by Koropatnick et al. (2004). Aside from pathogen recognition and elimination, the insect immune system must distinguish between, and respond differently to commensal and pathogenic microorganisms. This occurs via the sensing of danger/damage molecules (DAMPs), whether endogenous or exogenous. For example, it is already known that proteases secreted from microorganisms, or anomalous proteolytic activity in the hemolymph, can induce the activation of immune pathways in insects, leading to the production of antimicrobial molecules (Chamy et al., 2008). This paired system of sensing MAMPs and associated DAMPs may constitute a primary immune regulation mechanism in insects.

Matzinger (1994) was the first to state that danger signals are required to activate an appropriate defense against pathogens, and are a product of tissue or cell damage. Based on this theory, it has been suggested that tissues or cells of insects could also release danger signals and that the level of these signals should correlate with a damage threshold upon the establishment of infection (Cooper, 2010; Lazzaro and Rolff, 2011; Moreno-García et al., 2014). The idea that insects can recognize and react against these danger/damage molecules was in part proposed 45 years ago by Salt (1970). In his seminal paper, he anticipated that microorganisms damage host tissues of insects directly or indirectly by “… releasing abnormal if not toxic substances. An insect has need, then, for protection against foreign or abnormal particles of molecular size.” He mentioned that the defense is not only related to recognition of self or non-self, but also to damage: “Since the cells of each kind of insect react to most infections, but not to all, those cells must be able to distinguish the organisms to which they react from others which they do not molest” and called attention to the fact that the … “defence reaction must lie principally in its effect on alien parasites, and particularly on those that endanger the life of their host.”

During evolution, several molecules have arisen that have a function in sending information from one living cell to another. Salt (1970) anticipated that “… blood cells react to a negative characteristic, the absence of something; or that negative characteristic must be transformed into a positive stimulus.” He also pointed out that a non-self-entity “… would be expected to have an effect on some of those substances quite apart from any effect on cells ... If those altered plasma molecules stimulated the blood cells, they would provide the positive stimulus required.” In plants and animals a number of these intracellular molecules have been reported to stimulate the immune response when appearing at high concentrations in the extracellular space (reviewed in Heil, 2012). Hence, it is now considered that the immune capacities of insects rely not only on the discrimination of self and non-self, but also on a damage threshold that triggers the host “decision” to either eliminate or co-exist with microorganisms (Moreno-García et al., 2014).

Matzinger and Kamala (2011) and Medzhitov et al. (2012) proposed that tissues have an intrinsic ability to tolerate some degree of stress, damage, or malfunction. Likewise, Salt presumed that tissues must play an essential role in immunity and wound repair: “An organism attempting to infect the body of an insect (not merely its gut) must enter the haemocoel, if only to pass through it on the way to a particular tissue. Defence against such organisms can appropriately be deployed in the haemocoel, where the infection can be overcome, if the reaction is successful, before any other tissue or organ is harmed.” Finally, he implicated the sensitivity of tissues, the sensing of danger/damage molecules, and the relation of these two factors to defense: “Although these parasites ultimately cause dreadful damage, they are inserted with negligible injury; and the haemocytes are inactive against them because they are within the connective tissue covering the organ.” Therefore, in accordance with the ideas of Matzinger, Medzhitov et al. and Salt, in insects there must also be injury to tissues and this could be met by a variable damage tolerance. Then, for the maintenance of the integrity/morphostasis of a tissue in this animal group (see Cunliffe, 1997; Dembic, 2000), wound repair and final host survival depend on the recognition and sensing of self/non-self molecules, as well as the damage and immune response induced.

*Drosophila melanogaster* has been commonly used as a model for exploring the mechanisms of insect immune defense. Likewise, vector-borne mosquitoes, by virtue of their impact on human health, have also aroused interest and increased our comprehension of the molecular and cellular interactions between insects and the various developmental stages of pathogens/parasites. Although the danger/damage concept has now been incorporated into recent reviews, some aspects of damage caused by microorganisms in vector-borne arthropods have recently been neglected.

In the *Anopheles–Plasmodium* interaction, as the parasite passes through various stages of development it must penetrate (with associated damage) several physical barriers to reach the hemolymph and then the salivary glands: the peritrophic matrix, the midgut epithelial cell layer, and the basement membrane (BM). Much remains unclear about this process. For example, how are parasites able to develop and escape from the mosquito immune response? Given that the lifespan of the mosquito is not much longer than 30 days (Grieco et al., 2003; Hurd et al., 2005), and that parasite development takes, depending on the species of *Plasmodium*, from 15 to 25 days, how is it possible that the mosquito can transmit the parasite to humans? Moreover, what is the nature of the signals that activate the immune response against this parasite and associated damage? To answer these questions, we reassess the *Anopheles–Plasmodium* interaction by incorporating the danger/damage hypothesis and Salt’s assumptions. Knowledge about damage caused by parasites may help to understand the relevance of tissue damage, the immune response, and the origin and progression of infection in this insect–parasite interaction.

## THE PARASITE’S MULTIPLE DEVELOPMENTAL STAGES IN MOSQUITOES

*Plasmodium* is the parasitic protozoa that causes malaria. It possesses an apicoplast (a plastid-like organelle) and an apical complex, which are present in its three invasive stages. While in the vertebrate host the parasite is mainly intracellular, in the mosquito it is primarily extracellular, invading cells solely because of its need to cross tissue epithelia (Bannister and Sherman, 2009). After a mosquito ingests *Plasmodium* parasites (through infected blood meal), the arrested gametocytes mature and emerge from the erythrocytes within minutes. Motile microgametes fertilize macrogametes, and a zygote is formed. After 18–20 h the zygote differentiates into a motile invading stage called the ookinete, which migrates out of the blood bolus and crosses two barriers. The first one is the chitinous peritrophic matrix that is secreted by the midgut epithelial cells after each blood meal. The second is the midgut epithelium, a single cell layer surrounded by a thin and sparsely reticulated muscular tissue and the BM (Vlachou et al., 2006; Angrisano et al., 2012). The BM, an extracellular protein sheet surrounding tissues of animals, is composed primarily of laminin, collagen IV, and proteoglycans. There is a high homology in composition and function between the BM of invertebrates and vertebrates (Yurchenco and O’Rear, 1993).

**FIGURE 1:**
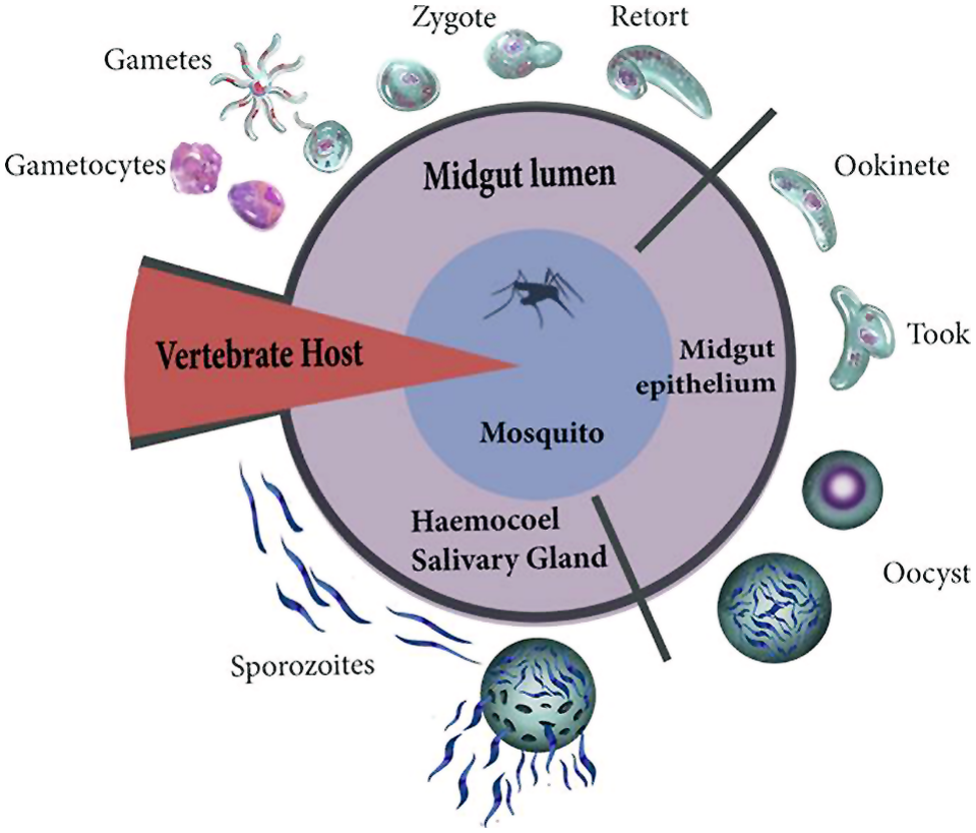
***Plasmodium* invasion starts with the entry of gametocytes and formation of male and female gametes.** The pronuclei of both gametes fuse for fertilization resulting in the formation of the zygote. The zygote then initiates its transformation (retort) into an ookinete. During invasion, the ookinete crosses the peritrophic matrix and reaches the luminal face of the epithelium. The ookinete differentiates (took forms) into a rapidly growing sporozoite-producing oocyst. The DNA of oocysts replicates forming 1000s of sporozoites that are released into the hemolymph. These sporozoites migrate to reach the salivary glands.

Once the ookinete reaches the BM (at 24–36 h post-feeding) it ceases its mobility, fuses with this membrane, and differentiates into a vegetative oocyst. The oocyst feeds on hemolymph components to grow from about 5 μm to as much as 50 μm in diameter during the following 12 days. The DNA of oocysts replicates from 8 to 10 times, forming 1000s of sporozoites (Canning and Sinden, 1973; Rosenberg and Rungsiwongse, 1991; Nacer et al., 2008) that are released into the hemolymph. These sporozoites migrate, either by gliding motility while adhered to the BM or more commonly by means of the open circulatory flow. In the latter case they pass through the tubular heart to reach the salivary glands. The sporozoite then crosses the BM and the salivary gland epithelial cells, staying in the lumen of this gland until the mosquito injects them with saliva when taking a blood meal (Rodriguez and Hernández-Hernández, 2004; Mueller et al., 2010). Maier et al. (1987) established five stages in the interaction of the malaria parasite with the host that could generate damage (**Figure 1**): (1) exflagellation and ookinete development; (2) epithelial damage caused by the penetration of ookinetes; (3) competition for host metabolic products by the growing oocyst; (4) sporozoite migration; and (5) penetration of salivary gland cells by sporozoites.

## THE MALARIA PARASITE TRANSITION IN MOSQUITOES, THE DAMAGE IT GENERATES, AND ITS ADAPTATIONS TO AVOID IT

### OOKINETE MIDGUT INVASION

The midgut plays a significant role in fluid and nutrient digestion and excretion, as well as in the synthesis and secretion of mucus, digestive enzymes, and the peritrophic matrix (Gooding, 1973; Hecker, 1977; Billingsley and Lehane, 1996). The latter tissue has direct contact with a wide variety of external stimuli such as the microbiota, the complex mix of dietary components, microorganisms that are ingested with the meal, by-products of digestion, pathogens, and toxins. This tissue establishes a selective barrier that actively absorbs nutrients to convert them into metabolites and store them, and provides a first line of defense against potentially harmful agents or pathogens (Gooding, 1973; Billingsley and Rudin, 1992). On the other hand, the continuity of the gut epithelium depends on the constant communication as well as the mechanical connections between its cells. These two factors establish a metabolic and electrical integration with impact on tissue homeostasis (Harvey and Blankemeyer, 1975).

During invasion, the ookinete crosses the peritrophic matrix and reaches the luminal face of the epithelium. It must subsequently adhere to these epithelial cells and invade them. Until recently there was an ongoing debate about whether the parasite takes an intracellular or intercellular route across the midgut epithelium. Those arguing in favor of an intercellular route presented evidence that the parasite was observed between the basolateral membranes of the epithelial cells. This occurs when the ookinete enters the epithelium in the regions where three epithelial cells join. However, it is now almost certain that the ookinete enters the epithelial cell, but without the formation of a parasitophorous vacuole, aided by perforin-like molecules (Angrisano et al., 2012). Ookinetes invade many cells on their way to the BM (although it is not clear why this happens), causing every invaded cell to enter into apoptosis (Han et al., 2000; Zieler and Dvorak, 2000; Vlachou et al., 2004).

It has been proposed that the apoptosis of cells and their consequent extrusion from the epithelium obliges ookinetes to migrate laterally to a neighboring cell to avoid being extruded. This idea is the so-called “time bomb” theory proposed by Han et al. (2000). Another model, denominated the “cellular treadmill” and proposed by Baton and Ranford-Cartwright (2005), suggests that ookinetes only move from the apical surface to the basal membrane of the epithelial cell, not laterally. But since neighboring cells fill the gap left behind by an apoptotic cell, an ookinete must invade these cells as well (Baton and Ranford-Cartwright, 2005).

Parasite invasion is a density-dependent process. Within a certain threshold, the lower the parasite burdens in the mosquito, the greater the transmissibility of the disease. For example, under laboratory conditions with the *Anopheles stephensi–Plasmodium berghei* model, the efficiency of producing oocysts begins to decrease when there are over 355 ookinetes per mosquito (Sinden et al., 2007). This is ascribed to the fact that the parasite damages the mosquito, and the energy is redirected to the immune response mounted against it (Tripet et al., 2008). Hence, the lesser the damage generated, the greater the overall fitness for both interacting species (Churcher et al., 2010). During the crossing of the mosquito midgut barriers, a further decrease in ookinete counts is observed. Despite the resulting low number of oocysts, each one is capable of forming 1000s of sporozoites, many of which are eliminated in the hemolymph by several factors (see Section “Sporozoite Invasion of the Hemocoel: Damage to the Basement Membrane, Tissue Protection, and Cellular Immunity”).

Although the invasion process induces damage, well adapted and less virulent parasites could have a selective advantage. Whitten et al. (2006) mentioned: “… because the junctional complexes at the apical surface are rigid, and their integrity is vital to the survival of the cell, the parasite may attempt the mechanically less demanding and damaging process of intracellular penetration of the epithelial cells.” Since leakage of the blood bolus into the hemolymph is probably a worse scenario than the parasite invasion itself, the ookinete may enter the cell primarily to leave the junctional complex intact (Whitten et al., 2006). Once the ookinete approaches the basal labyrinth of the epithelial cell being extruded, neighboring cells have already started to extend lamellipodia beneath the invaded cell. Thus there is no “hole” left behind by the extruded cell (Vlachou et al., 2004). Furthermore, the parasite seems to have a “hood” surrounding it, which is produced by lamellipodial extensions of the invaded cell itself. In the words of Vlachou et al. (2004): “Hood formation may be stimulated by the parasite and could represent a compromise that potentially hinders egress of the ookinete …” (at this point the ookinete is severely constrained) “… but also prevents draining of the invaded cell through the perforated plasma membrane. Draining would create a dangerous shunt between the enzyme-rich contents of the midgut lumen …” (that is, the epithelial cell contents that spill as a result of necrosis) “… and the open circulatory system of the mosquito, the haemolymph, just across the porous basal lamina.”

**FIGURE 2:**
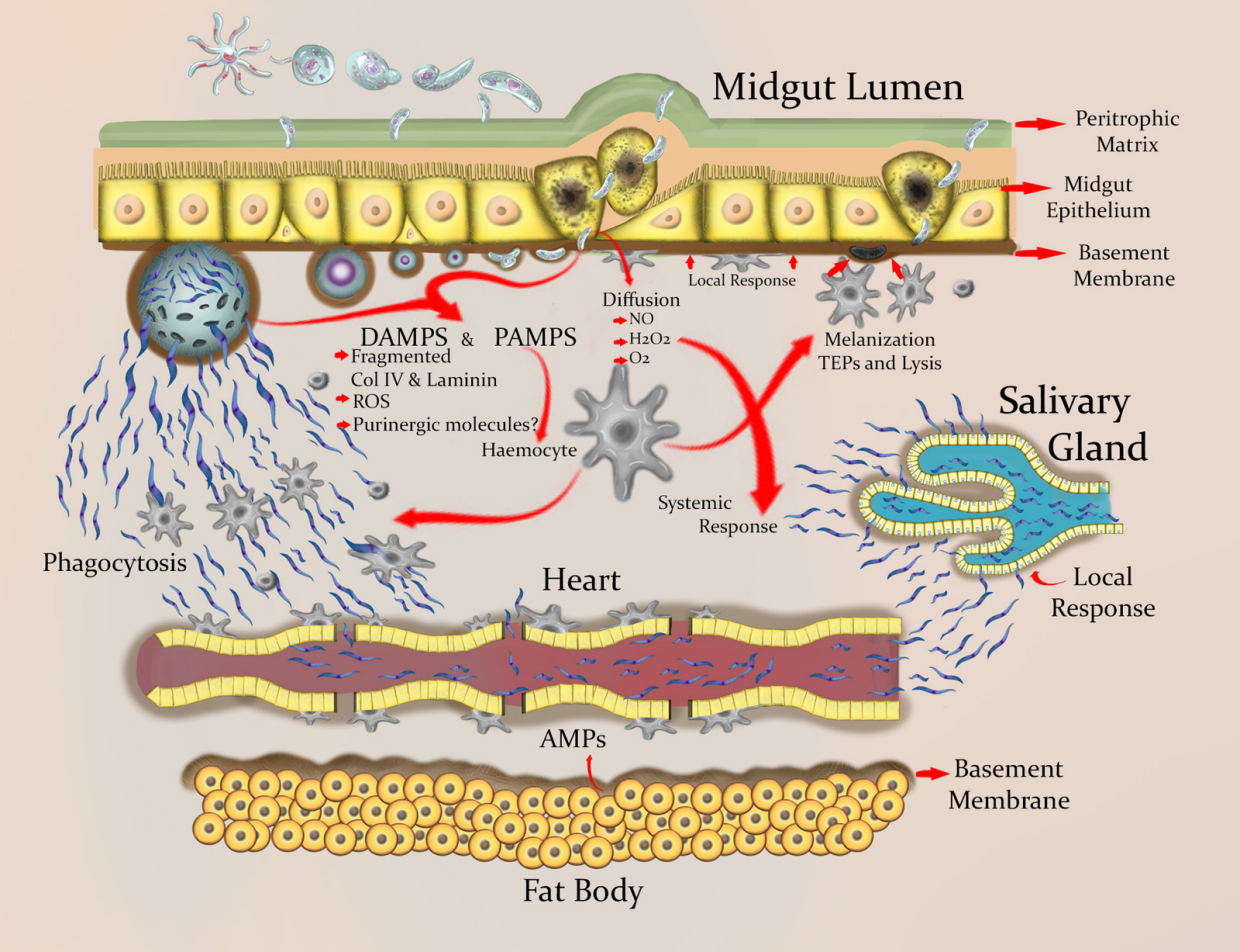
**Schematic view of the wounding and inflammation process in mosquito tissues during *Plasmodium* infection.** Ookinetes wound the peritrophic matrix and midgut epithelial cells, which leads to the loss of some of these cells. Damaged epithelial cells are extruded, followed by the differentiation of regenerative cells and the consequent proliferative regeneration. The dead cells release intracellular contents that may function as DAMPs. ROIs and adenosine-containing molecules appear to be essential endogenous signaling molecules needed for protection against danger. In *Anopheles* spp., genes involved in biological processes such as immune recognition, immune signaling pathways, and autophagy are expressed during a *Plasmodium* infection. This expression can be detected locally in the gut and systemically in body fat. ROIs limit parasite development and have signaling activity in various tissues during infection. The ookinete differentiates into an oocyst in the space between the BM and the basal side of the epithelium. In this stage the parasite is susceptible to being melanized and is exposed to TEPs, AMPs, PO cascade products, and ROIs. Sporozoites emerge in the hemolymph, and are to some extent limited by the differentiation and proliferation of hemocytes. However, hemocytes have another perhaps more important function during the stage of sporozoites, which is the ability to sense small-sized BM fragments and adhere to sites where the BM is damaged and to places with cell debris. Basal lamina components for wound repair are produced by circulating hemocytes. Migration of sporozoites through the hemolymph and the tubular heart is probably hindered by the accumulation of hemocytes in the ostia. Finally, sporozoites accumulate in the salivary gland of the mosquito, from where they are transmitted to a vertebrate host.

Ookinete invasion damages midgut epithelial cells and thus leads to cell death (Maier et al., 1987; **Figure 2**). However, this damage does not always have a detrimental effect on mosquito survival (Ferguson and Read, 2002). This suggests that adult female mosquitoes have an efficient mechanism for midgut repair and regeneration (Baton and Ranford-Cartwright, 2007). Following damage, epithelial cell extrusion is observed (Baton and Ranford-Cartwright, 2004) and regenerative cells differentiate into columnar cells, leading to regeneration of the mosquito midgut epithelium (Baton and Ranford-Cartwright, 2007). Interestingly, the plasma membrane of invaded epithelial cells seems compromised, and therefore intracellular contents are released (Zieler and Dvorak, 2000), The molecules of the intracellular contents, such as intracellular nucleotides, reactive oxygen species, extracellular purinergic molecules, nucleic acids, and heat shock proteins, could act as DAMPs, which when sensed by the innate immune system trigger an inflammatory response (**Figure 2**; see Section “ Is There Damage-Related Pathology and Inflammation in Plasmodium Infected Mosquitoes?”). Oxidative stress, bacterial infection, DNA damage, aging, and other factors cause apoptosis and damage to the enterocytes (Ayyaz and Jasper, 2013). However, these stressors also induce regenerative cell proliferation in the gut (Ayyaz and Jasper, 2013).

### THE MOSQUITO IMMUNE RESPONSE TO *Plasmodium*

Insects lack an adaptive immune response based on somatic generation and clonal expansion of specific immune cells (Du Pasquier and Flajnik, 1999). However, evidence shows that insects are indeed able to enhance immunity to an infection after a first exposure (providing resistance, better immunocompetence, and survival advantages), an advantage that can persist across generations (Little et al., 2003; Little and Kraaijeveld, 2004; Kurtz, 2005; Moret, 2006; Pham and Schneider, 2008; Rodrigues et al., 2010; Tidbury et al., 2011; Contreras-Garduño et al., 2014). This mechanism is known as immune priming and is considered to be similar to vertebrate adaptive immunity (Little et al., 2005; Litman and Cooper, 2007).

In insects, both cellular and humoral components of the immune response contribute very significantly to resistance against microbial infection (Hoffmann et al., 1999). The insect immune system consists of a variety of responses, the first occurring in the epithelial barriers (epidermis, intestinal, and tracheal network). From these organs, the response may spread systemically via the hemolymph, an open circulatory system that fills the hemocoel. A systemic response primarily involves fat body (the main producer of the hemolymph proteins) and hemocytes (involved in phagocytosis, nodule formation, and encapsulation). These humoral and cellular components allow for a rapid and efficient insect immune response upon infection.

Overall, the defense processes of insects can be divided into two main stages: recognition and response. Each of these are connected by signaling pathways and regulated by modulating elements (Christophides et al., 2004). Molecules involved in recognition have been grouped as soluble receptors and those located in cell membranes. Recognition is carried out by proteins that recognize peptidoglycan and Gram-negative bacteria, thioester bond-containing proteins (TEPs), and scavenger receptor type lectins. After the foreign element is recognized, a signal is transmitted to the cell nucleus for activation of target genes. These pathways include the Toll and IMD systems, and in the case of mosquitoes a third one named the JAK/STAT pathway. Modulation of the response is mediated by extracellular serine protease cascades, which are regulated by specific inhibitors (serpins). Defense responses include the activation of a large number of genes, and characteristic effectors include antimicrobial peptides (AMPs), melanization dependent on the phenoloxidase (PO) cascade, and apoptosis-related genes (**Figure 2**). Regarding the immune response to intracellular infectious agents such as viruses, it is speculated that the Toll and JAK/STAT pathways are involved (Sanders et al., 2005; Rivkin et al., 2006), but the underlying signaling mechanism is still unknown.

When *Anopheles gambiae* is challenged with different pathogens or infected with *Plasmodium* parasites, at least 200 genes are differentially expressed (Dimopoulos et al., 2002). The expression of immune markers during *Plasmodium* infection can be detected locally in the gut and systemically in body fat. The latter is considered the main tissue that produces immune proteins released into the hemolymph (Dimopoulos et al., 1998, 2002). Overexpression of immune genes during infection begins when parasites are present in the mosquito midgut (Luckhart et al., 2003). Recently, Martinez-Barnetche et al. (2012), explored different tissues of the adult female of *An. albimanus* to identify the transcriptome related to a *Plasmodium* infection. They identified protein-coding transcripts involved in biological processes such as immune-recognition, immune signaling pathways, insecticide resistance, and autophagy, all of which are related to a *Plasmodium* infection. The analysis of the midgut of *An. albimanus* infected with *P. berghei* revealed the proteins that are differentially expressed during this immune challenge (Serrano-Pinto et al., 2010). The two most common functional classes are immunity/defense (34.78%) and blood digestion proteases (15.21%).

There is evidence that the production of nitric oxide (NO) in the midgut epithelium of the mosquito *An. stephensi* limits the development of *P. berghei* (Luckhart et al., 1998; Han et al., 2000; Luckhart and Li, 2001). The midgut of *An. pseudopunctipennis* responds similarly by producing NO in the presence of bacteria, yeasts and *Plasmodium* (Herrera-Ortíz et al., 2004), while that of *An albimanus* produces reactive oxygen species (e.g., the superoxide anion) that are toxic to *P. berghei* ookinetes (Lanz-Mendoza et al., 2002). These observations suggest the importance of various tissues during the immune response of these mosquitoes.

It is possible that the immunological response mounted by a mosquito during an ookinete invasion is not the only mechanism limiting the density of parasites. The malaria parasite itself might contribute to the regulation of its density in mosquitoes by means of programmed cell death (PCD; Pollitt et al., 2010; Matthews et al., 2012). This possibility together with the apoptosis of midgut cells that are invaded by ookinetes (Zieler and Dvorak, 2000), which are extruded from the epithelium into the lumen of the midgut (Han et al., 2000), suggests a certain limit for the number of ookinetes than can pass through the midgut before the physiology of the intestinal tissue is compromised. Therefore, the parasite might develop the counterintuitive strategy (in terms of intraspecific competition, but not in terms of evolutionary ecology) of regulating its population density in order to minimize the damage to the host. This behavior has been observed in the blood stages (Mutai and Waitumbi, 2010) and the mosquito stages of the parasite (Al-Olayan et al., 2002), although the latter needs to be considered critically since the ookinetes are not clones, because they are produced after sexual reproduction. If this hypothesis holds, it will emphasize the importance for the parasite of avoiding an overactive immune response of its host to avoid damage to the latter.

### THE MALARIA PARASITE INVISIBILITY CLOAKS

Reaching the BM is only half the trajectory the malaria parasite must complete to be transmitted to the next vertebrate host. Between the BM and the basal side of the epithelium surrounded by the hemolymph, this parasite enters a vegetative stage of 10–15 days in which the ookinete differentiates into a rapidly growing sporozoite-producing oocyst. In this period the parasite is melanized by hemocytes (Whitten et al., 2006) and exposed to soluble immune proteins like the TEPs (Marois, 2011), antimicrobial peptides, PO metabolites, and reactive oxygen and nitrogen species (Luckhart et al., 1998; Lanz-Mendoza et al., 2002; Herrera-Ortíz et al., 2004; Herrera-Ortiz et al., 2011; Clayton et al., 2014). Nevertheless, there is evidence of strategies employed by the parasite to render itself invisible to the immune response. Arrighi et al. (2005) have shown than the ookinetes, as well as the oocyst, are coated with laminin derived from the mosquito. Furthermore, when these researchers silenced the laminin gene with dsRNA, the transmission of the parasite was hampered (Arrighi et al., 2005). A related study found that Sephadex molecules covered with laminin derived from mice, *Drosophila* cells, or the parasite surface protein PgS28 (which binds laminin) were less melanized in comparison with non-coated Sephadex beads when inoculated into the hemocoel of *Aedes aegypti* (Warburg et al., 2007). However, these experiments do not provide definitive proof for this “mask theory” because parasite development may have been hampered for reasons other than a masking mechanism.

Interestingly, Nacer et al. (2008) found laminin to be present not only in the oocyst capsule but also in the membrane of the developing sporozoites within the oocysts. This suggests that the oocyst covers the sporozoites with laminin during their formation to prevent their melanization upon release to the hemocoel. The finding that the oocyst has transglutaminase, which could cross-link mosquito proteins – including laminin – to the capsule (Nacer et al., 2008; Smith and Jacobs-Lorena, 2010), suggests that the components of the oocyst capsule may be derived from parasite and mosquito proteins. However, this question requires further research.

Finally, there is recent evidence that PSF47, a surface ookinete protein, somehow inhibits the expression of the epithelial heme peroxidase (HPX2) and NADPH oxidase 5 (NOX5). The expression of these two proteins leads to the nitration of ookinete surface proteins, which could cause the parasite to be tagged for TEP1 mediated lysis or melanization when reaching the BM. However, this applies to some parasite-mosquito species (or strains) reflecting a local arm race between the parasite and the mosquito (Molina-Cruz et al., 2013; Philip and Waters, 2013).

### SPOROZOITE INVASION OF THE HEMOCOEL: DAMAGE TO THE BASEMENT MEMBRANE, TISSUE PROTECTION AND CELLULAR IMMUNITY

Once the oocyst is completely mature, sporozoites emerge in the hemolymph and invade the salivary gland. Sporozoites accumulate in the salivary ducts of the glands and from there are transmitted to a mammalian host. From 1000s of sporozoites released, only 10–25% manage to invade the salivary glands (Sinden and Billingsley, 2001). What are the mechanisms that could explain this drastic reduction in sporozoites? Some authors have suggested a possible interaction between cellular and humoral molecules (e.g., Pinto et al., 2008), such as AMPs, complement-like proteins and molecules produced during the melanization process (PO cascade; e.g., Dimopoulos et al., 2002; Clayton et al., 2014).

Hemocytes are strongly activated during the ookinete and oocyst stages, and the cellular response is responsible for conferring immune memory against the latter stage (Rodrigues et al., 2010; Ramirez et al., 2014). Hemocytes count increase and also are induced to differentiate during the sporozoite stage as well (Hillyer et al., 2007), and are partly responsible for limiting sporozoite circulation in the hemocoel (Hillyer et al., 2003). However, phagocytosis and melanotic encapsulation is weak (Hernández-Martínez et al., 2002). Even some TEPs, which are produced by the hemocytes and efficiently bind to and mediate the killing of ookinetes and oocysts, do not bind to sporozoites (Blandin et al., 2004). Why do the morphology and number of hemocytes change during the sporozoite stage if this immune molecule responds only weakly against sporozoites? There are two possible and related explanations: (1) the cellular response becomes activated mainly for BM wound repair, and (2) the immune response is aimed to protect tissues (e.g., heart) with a lower intrinsic ability to tolerate damage.

Sinden (1974) reported the presence of small holes (0.25–0.65 μm in diameter) in the oocyst that were limited to areas of about 15 μm. For sporozoites to be released from the oocyst wall, the BM must be ruptured or degraded (or at least some continuity must be lost), however, the partially disrupted BM did not prevent sporozoites from being released (Sinden, 1974; Sinden and Strong, 1978; Meis et al., 1992). Since the BM separates midgut epithelial cells from the hemolymph and hemocoel, damage to this tissue must heal quickly. Then, immediately after sporozoite invasion into the hemolymph, BM wound healing must sets in to isolate the hemocoel and prevent intrusion of opportunistic bacterial via co-penetration.

Lackie (1988) proposed that an important role of hemocytes is the formation and repair of the BM and wound sealing, where the numbers and subpopulations of hemocytes vary in response to the extent of damage and introduction of foreign material. She (like Salt) pointed out that the immune system discriminates degrees of difference from self, a task that requires soluble factors derived from the wounded area. These factors control the resulting hemocytic response (Lackie, 1988). In *Drosophila*, sterile damage in cuticular basal lamina (which is part of the BM) promotes hemocyte differentiation and an increment in hemocyte counts (Márkus et al., 2005). Related to this idea of a damage-induced immune response, Nayar and Knight (1995), showed that cuticle wounding (with an injection of saline solution) enhanced the cellular encapsulation and melanization responses against a nematode. Moreover, hemocytes adhere to sites where the BM is damaged, but not to an intact BM (Rizki and Rizki, 1980). Meanwhile, circulating hemocytes are rapidly recruited to the site of damage. The first arriving hemocytes bind to sites where cell debris exist (Babcock et al., 2008). In *An. gambiae* basal lamina components are produced by circulating hemocytes, which secrete them onto cell surfaces within the hemocoel (Pastor-Pareja et al., 2008). Thus, it is likely that degraded components of the BM serve as DAMPs (**Figure 2**).

In agreement with this idea, Altincicek and co-workers (Altincicek and Vilcinskas, 2006; Altincicek et al., 2007) have proved the ability of the insect to sense small-sized collagen IV fragments. These fragments induce the activation Toll and IMD immune pathways (in body fat), leading to the synthesis of antimicrobial molecules (Altincicek and Vilcinskas, 2006). In *Anopheles* mosquitoes, the anti- *Plasmodium* defense is principally controlled by these two pathways (Clayton et al., 2014). Therefore, during sporozoite release and invasion, it is possible that the BM is damaged and that the recognition of free fragments induces hemocyte recruitment. On the other hand, the increased number of circulating hemocytes could be more a consequence of the activation of immune pathways related to the sensing of the parasite and DAMPs (Pastor-Pareja et al., 2008).

The fact that in mosquitoes the cellular response is activated against ookinetes and oocysts, and that this elicits an enhanced immune response when re-exposed (immune priming), could be related with the capacity of the immune system to adjust its primary response after exposure to danger signals (“memory of danger,” *sensu* Noble, 2009). Foley (1978) suggested that if hemocytes respond to a BM disruption, their adherence to the BM leaves fewer circulating hemocytes to respond to the migrating sporozoites. In mosquitoes, immune priming has been supported by experimental approaches (Rodrigues et al., 2010; Contreras-Garduño et al., 2014; Ramirez et al., 2014). Thus, danger signals may induce immune responses that protect the injured tissue from opportunistic infections. Tissues have an intrinsic ability to tolerate some degree of stress, damage, or malfunction. Therefore, the cellular response elicited during midgut invasion and oocyst establishment implies communication between the midgut epithelium, hemocytes and other tissues that have not yet had direct interaction with the pathogens (see Section “Danger Molecules”). Subsequently, if pathogens get access to the hemocoel and tissues therein, an enhanced primed response may limit damage in these tissues.

In *An. gambiae*, sessile hemocytes exist in a relatively high proportion (∼25%). They have an efficient phagocytic activity and are mainly attached to the trachea or the ostia of the heart, which could function as a protective strategy to limit potential pathogen dispersal through hemocoel (King and Hillyer, 2013). In insects, the circulatory system consists of a dorsal vessel that allows for the transport of hormones, waste materials and nutrients, as well as immune surveillance by the circulation of molecules and hemocytes (Klowden, 2008). When mosquitoes become infested with sporozoites, there is an induction of phagocytic hemocyte aggregation in the dorsal vessel (pericardial region; King and Hillyer, 2012). Hemocyte recruitment and attachment along the dorsal vessel and pericardial cells (nephrocytes; see Hernández-Martínez et al., 2013) may serve as an efficient strategy for eradicating some sporozoites, but perhaps more importantly for limiting the entrance of other pathogens through a wound in the BM (e.g., Matuschewski, 2006) and protect and maintain tissue integrity.

Injury to the BM and the probability of further damage to other tissues could be considered an activator of the immune response, which would represent evolutionary changes similar to those found with immune responses against pathogens in insects. The initial molecular factors (and/or mediators) of damage responses use the same immune cells and pathways as the immune response to invasive parasites, and could stimulate unique types of reactions when damage/danger and parasites are sensed at the same time (e.g., Chamy et al., 2008). If this dual system does in fact exist, the activation of the immune response not only depends on the type of pathogen, but also on the presence, intensity, and probability of damage.

## DANGER MOLECULES

### REACTIVE OXYGEN INTERMEDIATES (ROI)

Medzhitov et al. (2012) suggested that tissues differ in their ability to tolerate stress and damage. The communication among tissues could be useful if some tissues have low or no renewal capacity. Therefore, the response observed during early *Plasmodium* development suggests the interaction of molecules of the midgut epithelium with other cells and tissues that have not yet had direct contact with the parasite, thus mediating a systemic immune response to wound prevention.

Inducible DAMPs released by metabolically stressed cells are reactive oxygen intermediates (ROS), and these molecules have been found to activate NF-κB in flies and mammalian cells (Gallucci and Matzinger, 2001). In mammals, the effects of reactive oxygen intermediates (ROIs) have been reported in several aspects of innate and adaptive immune responses (Rutault et al., 1999). Hence, endogenous factors influence the maturation of immune cells (Bagley et al., 2006). In insects the role of free radicals in the elimination of pathogens is well known, but their possible function as damage signals for activating the immune response is not as well understood. Recently it has been documented that the systemic wound response (SWR) in *Drosophila* is dependent on a serine protease called Hayan (Nam et al., 2012). This protease is able to sense integumental wounds and activates the phenoloxydase cascade (PPO). It is also known that during the activation of the PPO cascade several ROS are produced (Nappi and Vass, 1993), including hydrogen peroxide (H_2_O_2_). The latter molecule leads to the activation of JNK-dependent cytoprotecive program in neuronal tissues (Nam et al., 2012). As can be appreciated, these findings provide a link between wound response and the nervous system.

In *An. gambiae*, H_2_O_2_ levels increase dramatically after a blood meal, probably due to an increase in the metabolic processes associated with blood digestion and oogenesis (DeJong et al., 2007), as well as the defense mechanisms induced by bacterial growth in the midgut after blood-feeding. On the other hand, it has been suggested that H_2_O_2_ contributes to mosquito defenses. Levels of H_2_O_2_ significantly increase in an *An. gambiae* malaria refractory strain (compared to the susceptible strain) after an infected blood meal (Kumar et al., 2003). Also, in the presence of L-DOPA, the hemolymph and midgut of anopheline mosquitoes generate the superoxide anion, which is toxic to *P. berghei* ookinetes (Lanz-Mendoza et al., 2002; Kumar and Barillas-Mury, 2005). NO is produced in the *Anopheles* midgut during a *Plasmodium* infection (Dimopoulos et al., 1998; Luckhart et al., 1998; Herrera-Ortíz et al., 2004), and this molecule and its metabolites limit parasite development (Luckhart et al., 1998; Peterson et al., 2007). In *Drosophila*, infection with Gram-negative bacteria induces the expression of nitric oxide synthase (NOS). The inhibition of NOS diminishes larval survival in the face of Gram-negative bacterial infection. In *An. Gambiae*, midgut epithelial cells also respond to *Plasmodium* ookinete invasion by inducing the expression heme peroxidase 2 (HPX2), which in combination with NOS mediates parasite nitration (Kumar et al., 2004; Oliveira et al., 2012).

The involvement of H_2_O_2_ in the activation of the acute phase of the immune response via NF-κB is well documented (Gloire et al., 2006). In *Hyalophora cecropia*, the immunoresponsive factor (CIF) could be activated by H_2_O_2_ (Sun and Faye, 1995), and 5-*S*-GAD (N-β-alanyl-5-*S*-glutathionyl-3,4-hydroxyphenylalanine) from *Sarcophaga peregrina* produces H_2_O_2_ and is an activator of NF-κB (Natori, 1998). Additionally, NO produced during infection is involved in signal transduction for the expression of immune response genes. On the other hand, the inhibition of NOS activity prevents the induction of diptericin (Nappi et al., 2000; Foley and O’Farrell, 2003). Exogenous NO induced the production of this AMP in uninfected *Drosophila* larvae (Foley and O’Farrell, 2003), and the injection of an NO donor induced the expression of the cecropin B gene in *Bombyx mori* (Imamura et al., 2002).

It has been reported that the expression of AMPs is induced by the exogenous addition of NO and H_2_O_2_ in the midgut and abdominal tissue in *An. albimanus* infected with the rodent malaria parasite *P. berghei* (Herrera-Ortiz et al., 2011). This observation indicates that NO and H_2_O_2_, produced in the mosquito midgut during infection, might function as signals for the activation of the mosquito systemic immune response. On the other hand, midgut infection induced an increase in the expression of the three AMPs in both the midgut and abdominal tissue, while NO and H_2_O_2_ were present in the hemolymph. The induction of AMPs in abdominal tissues during midgut infection indicates communication between the midgut cells and abdominal tissue that has not yet had direct contact with the parasites. NO and H_2_O_2_ are an important part of this communication.

NOS induction may also occur through the generation of ROI. The upstream sequence of the NOS gene from *An. stephensi* has putative inflammatory responsive elements, including NF-κB (Luckhart and Rosenberg, 1999), and several lines of evidence indicate that ROI, in particular H_2_O_2_, are secondary messengers or activators of NF-κB (Nappi et al., 1995; Nappi and Vass, 1998; Mercurio and Manning, 1999). L-DOPA has been reported to induce ApNOS, but this induction could be aborted by catalase, indicating the participation of H_2_O_2_ in the process. Although no direct measure of H_2_O_2_ was made during the induction of ApNOS with different microorganisms, it has been observed that the midgut of *An. albimanus* mosquitoes, when inoculated by enema with *P. berghei* ookinetes, produced higher ^-^O_2_ than the midgut of mosquitoes inoculated with RPMI (Lanz-Mendoza, unpublished results). These observations indicate that malaria parasites may elicit ROI production in the cells of the midgut. Host DAMPs are released during stress and this triggers inflammation, which implies a potential danger to the host. ROI molecules could function as damage signals that activate the mosquito systemic immune response.

### PURINERGIC MOLECULES

Most living cells, tissues, and organisms have some form of sensitivity to purinergic molecules, such as ATP, AMP, ADP, and adenosine. The pervasive presence and abundance of all these molecules required their recruitment for intercellular signaling. ATP has an essential role in the mediation of pain, the activation of immune cells, the communication among nerve cells, and, importantly, the activation of the immune response (Trautmann, 2009; Burnstock and Verkhratsky, 2012). It is known that ATP-mediated signaling is used as a proxy for cell damage and the release of the intracellular content (e.g., Cook and McCleskey, 2002; Ivison et al., 2011; Zeiser et al., 2011).

Insects use purinergic molecules as phagostimulants (Friend and Smith, 1977) for energy metabolism and neuronal activity (Burnstock and Verkhratsky, 2009). In *Manduca sexta*, hemocytes hydrolyze extracellular ATP, and this activity increases when bacterial lipopolysaccharide is present (Meyer-Fernandes et al., 2000). Intriguingly, while ATP receptors (P2X) have been reported for some invertebrate groups, vertebrates and plants, non-homologous receptors have been found in insects (Fountain and Burnstock, 2009). The purinoreceptors in insects are adenosine receptors (AdoRs), and these respond to adenosine but not ATP (Trautmann, 2009). In this sense insect AdoRs are divergent from the mammalian AdoRs, but they both share the region for adenosine binding (Dolezelova et al., 2007).

Adenosine is a component of adenine nucleotides and can be generated through the release and degradation of ATP by adenosine deaminase (ADA), or by RNA degradation following cell death (Hirschhorn and Candotti, 2007). In vertebrates, extracellular adenosine has been proposed as an immediate sign of tissue damage (reviewed in Sitkovsky and Ohta, 2005). In insects, the role for adenosine has been suggested as a local paracrine and autocrine homeostatic regulator, indicating an evolutionarily well-conserved homeostatic mechanism (Dolezelova et al., 2007). In *Drosophila,* different cell types respond to extracellular adenosine and locally regulate its levels (Zurovec et al., 2002). Novakova and Dolezal (2011) reported that ADA expression is induced in encapsulating hemocytes, and that this can be correlated with tissue damage, the activation of Toll and JAK/STAT pathways, and the melanization of intrahemocoelic parasitic eggs (deposited by wasps into host hemocoel).

In *Anopheles* mosquitoes, the study of purinergic molecules and receptors is uncommon. Genes belonging to the adenosine deaminase-related growth factors (ADGF), ADA-like (ADAL), and AMP deaminase (AMPD) families have been reported for *An. gambie*. These families share ADA activity and an evolutionary relationship, suggesting an orchestrated control of adenosine levels (Maier et al., 2005). In mosquitoes (as well in other insect groups) it is unclear how extracellular purinergic molecules exert immune responses. Nonetheless, the occurrence of damage or death of midgut cells could lead to the release of this kind of molecule, which may activate receptors on adjacent cells. Adenine-containing molecules appear to be essential endogenous signaling molecules in mosquitoes, providing protection against danger in these insects just as they do in plants and vertebrates.

## IS THERE DAMAGE-RELATED PATHOLOGY AND INFLAMMATION IN *Plasmodium* INFECTED MOSQUITOES?

Ross (1902) was the first to observe the presence of “black spores” in infected mosquitoes (which correspond to parasites killed by encapsulation and melanization), leading to the conclusion that an infection was occurring in mosquito tissue, and that the insect responded to the parasite by encapsulating it. Pathological signs and symptoms in insects also manifest themselves as changes in coloration, abnormalities in movements and responses to stimuli, digestive disorders, and incapability to feed or reproduce (Tanada and Kaya, 1993).

Ferguson and Read (2002) concluded that in almost every case the parasite reduces the fitness of the mosquito. They described five effects in a mosquito infected with *Plasmodium*: (1) tissue damage and increased susceptibility to other infections, (2) a reduction in the levels of digestive enzymes, indicating a physiological change, (3) resource depletion (lower concentrations of free amino acids in the hemolymph, reflecting an increase in nutrient consumption), (4) an immune response, meaning that energy is directed to defense instead of being used for growth and reproduction, and (5) behavioral changes, such as the increased time required for feeding and the increased frequency of probing, penetration and feeding. A possible mechanism that could explain changes in feeding behavior is the decreased activity of salivary apyrase, a platelet inhibitor. Furthermore, the work of Rossignol et al. (1984) associated the decrease of salivary apyrase with the presence of fine lesions in the salivary gland (see Ferguson and Read, 2002). The virulence of *Plasmodium* depends on its mechanisms of invasion, which are responsible for the degree of damage done to the mosquito (Maier et al., 1987).

Historically, tissue damage has been related to redness, swelling, heat, and pain, currently known as the Celso’s cardinal signs of inflammation. Virchow (1863) mentioned a fifth cardinal sign – functio laesa, or a loss of function of the damaged tissue. Histopathological studies of inflammation have almost invariably been performed on vertebrates. However, the first studies that revealed the essential components of the complex phenomenon of inflammation were done on invertebrates (Metchnikoff, 1893). Based on the Darwin–Wallace conception of natural selection, the biological theory of Pasteur and the theory of cellular pathology of Virchow, Metchnikoff (1893) suggested that real inflammation, that which has been preserved along the phylogeny, occurs without heat and redness.

As in vertebrates, inflammation in invertebrates is produced by insults or lesions to cells and tissues, leading to the release of humoral substances that promote this immune response and to the migration of cells in the serum. The effect of inflammation is wound repair, replenishment of damaged cells, and the phagocytosis of dead cells (Metchnikoff, 1893). However, some of the soluble pathogenic and molecular components in the serum do not induce inflammation directly. Rather, they are associated with the signaling pathways of receptors on a cell surface that drive the cell to self-destruction. In turn, cell death regulates inflammation by releasing cell components or derivatives that alter the function of other cells, driving them to death or survival (Wallach et al., 2014). This is relevant presently because the interaction between *Plasmodium* and a mosquito leads to cell death in both organisms (Hurd et al., 2006). Studies designed to comprehensively evaluate potential regulatory effects of cell death could contribute to the understanding of this complex interaction.

## PERSPECTIVES

The *Anopheles* mosquito has become an important model for the study of how invertebrate immunity functions. Moreover, studying the development of *Plasmodium* in mosquitoes provides a significant opportunity to link tissue damage and the immune response. However, few studies have been conducted with molecules involved in signaling or tissue damage, molecules that may participate in activation and regulation of the immune response. Molecules involved in non-self recognition, while those that recognize damage-related molecular patterns (DAMPs) trigger signaling pathways that mobilize cellular and humoral defenses. The study of these three types of molecular patterns and their recognition will continue to involve ecologists, evolutionists, mosquito physiologists, and molecular immunologists. We encourage researchers to consider and incorporate Matzinger’s danger/damage hypothesis and George Salt’s injury assumptions when studying other insect–pathogen interactions. Future studies of the mechanistic attributes of damage produced during the pathogen–mosquito interaction could provide valuable information for understanding the activation and efficiency of the immune response under natural conditions.

Genomic and bioinformatics approaches have produced massive assemblages of information related to insect immunology in only a few years. We can expect that these data will provide a framework for the interpretation of the damage/immune response that could not have been inferred from studying the individual components in isolation. This kind of approach has been applied to comprehend the complex biological processes of wound healing (Aderem and Smith, 2004; Vodovotz, 2006), as well as host–pathogen interactions (Forst, 2006).

## Conflict of Interest Statement

The authors declare that the research was conducted in the absence of any commercial or financial relationships that could be construed as a potential conflict of interest.
